# Immunogenicity of DNA Vaccines Encoding Simian Immunodeficiency Virus Antigen Targeted to Dendritic Cells in Rhesus Macaques

**DOI:** 10.1371/journal.pone.0039038

**Published:** 2012-06-12

**Authors:** Matthias Tenbusch, Ralf Ignatius, Godwin Nchinda, Christine Trumpfheller, Andres M. Salazar, Katharina Töpfer, Ulrike Sauermann, Ralf Wagner, Drew Hannaman, Klara Tenner-Racz, Paul Racz, Christiane Stahl-Hennig, Klaus Überla

**Affiliations:** 1 Department of Molecular and Medical Virology, Ruhr-University Bochum, Bochum, Germany; 2 Institute of Tropical Medicine and International Health, Charité – University Medicine of Berlin, Berlin, Germany; 3 Laboratory of Cellular Physiology and Immunology, The Rockefeller University, New York, New York, United States of America; 4 Oncovir Inc., Washington, D.C., United States of America; 5 Unit of Infection Models, German Primate Center, Göttingen, Germany; 6 Geneart, Regensburg, Germany; 7 Ichor Medical Systems, San Diego, California, United States of America; 8 Bernhard Nocht-Institute for Tropical Medicine, Hamburg, Germany; Emory University School of Medicine, United States of America

## Abstract

**Background:**

Targeting antigens encoded by DNA vaccines to dendritic cells (DCs) in the presence of adjuvants enhances their immunogenicity and efficacy in mice.

**Methodology/Principal Findings:**

To explore the immunogenicity of this approach in non-human primates, we generated a single chain antibody to the antigen uptake receptor DEC-205 expressed on rhesus macaque DCs. DNA vaccines encoding this single chain antibody fused to the SIV capsid protein were delivered to six monkeys each by either intramuscular electroporation or conventional intramuscular injection co-injected or not with poly ICLC, a stabilized poly I: C analogue, as adjuvant. Antibodies to capsid were induced by the DC-targeting and non-targeting control DNA delivered by electroporation while conventional DNA immunization at a 10-fold higher dose of DNA failed to induce detectable humoral immune responses. Substantial cellular immune responses were also observed after DNA electroporation of both DNAs, but stronger responses were induced by the non-targeting vaccine. Conventional immunization with the DC-targeting DNA at a 10-fold higher dose did not give rise to substantial cellular immune responses, neither when co-injected with poly ICLC.

**Conclusions/Significance:**

The study confirms the potent immunogenicity of DNA vaccines delivered by electroporation. Targeting the DNA via a single chain antibody to DEC-205 expressed by DCs, however, does not improve the immunogenicity of the antigens in non-human primates.

## Introduction

**Figure 1:**
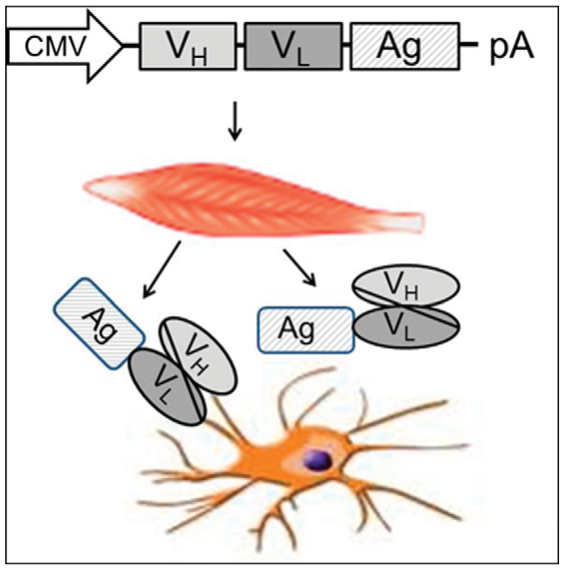
Principle of targeting of antigens encoded by DNA vaccines to DCs. The coding region of variable heavy (V_H_) and light (V_L_) chains of antibodies to uptake receptors of DCs are fused in frame to the open reading frame of the antigen. After delivery of the DNA vaccine, transduced cells of the immunized individual produce and secret a single chain antibody to the uptake receptor coupled to the antigen. Binding of the single chain to the uptake receptors should increase uptake and presentation of the antigen by the DCs.

DNA immunization is a promising vaccine platform with potential applications in prevention and treatment of infectious diseases and cancer. A number of different strategies are currently explored in more than 40 clinical trials to improve DNA vaccination (reviewed in [1]). One approach to improve the immunogenicity and efficacy of DNA vaccines is the targeting of the encoded antigen to molecules expressed by dendritic cells (DCs) such as DEC-205 (CD205) (Fig. 1). Notably, co-injection of DEC-205-targeted protein antigens with poly I: C or its analogue, poly ICLC that is stabilized against serum nucleases, which both bind to the innate pattern recognition receptors, Toll-like receptor 3 (TLR3) and melanoma differentiation-associated gene-5 (MDA-5) [2], [3], leads to increased antigen-specific T cell and B cell responses in mice [4]–[6] and non-human primates [7]–[9]. Injection of DC-targeted antigens in the absence of adjuvants, however, induces initial T cell proliferation, but this is not followed by strong CD4^+^ and CD8^+^ effector T-cell responses due to peripheral deletion, tolerance and/or induction of regulatory T cells [10]–[16].

Consistent with the results observed with the injection of DC-targeted proteins without adjuvants, we have also observed reduced immune responses after conventional intramuscular immunization with DNA encoding DC-targeted antigens in comparison to non-targeting DNA vaccines in mice [17]. In contrast, in the presence of TLR ligands, the immunogenicity of DC-targeting DNA vaccines was higher than that of the non-targeting control. Similarly, delivery of a DNA vaccine encoding DEC-205-targeted HIV Gag to mice by electroporation enhanced the efficacy of DNA vaccination in the absence of additional adjuvants [18]. In this situation, the strong inflammatory response known to be induced by intramuscular electroporation [19] might have overcome the requirement for other co-stimulatory signals.

The potent enhancement of antigen uptake by DCs and the ease of production of DNA vaccines would allow rapid testing of the immunogenicity of DEC-205-targeting DNA vaccines in humans. However, we felt that prior to advancing this approach into clinical trials, the immunogenicity of such immunization protocols should be evaluated in non-human primates. We therefore constructed and characterized a single chain antibody to the DEC-205 receptor of rhesus macaques and explored the immunogenicity of DNA vaccines encoding a fusion protein between the single chain antibody and the SIV p27 capsid antigen in this primate species. To evaluate the effect of DC-targeting, the targeting vaccine and a non-targeting control DNA were delivered by intramuscular electroporation and the SIV-specific cellular and humoral immune responses were compared. Additionally, we determined the impact of the application of poly ICLC as adjuvant on the immunogenicity of DC-targeting during conventional DNA immunization.

## Results

### Construction and characterization of single chain antibody to DEC-205 of rhesus macaques

**Figure 2:**
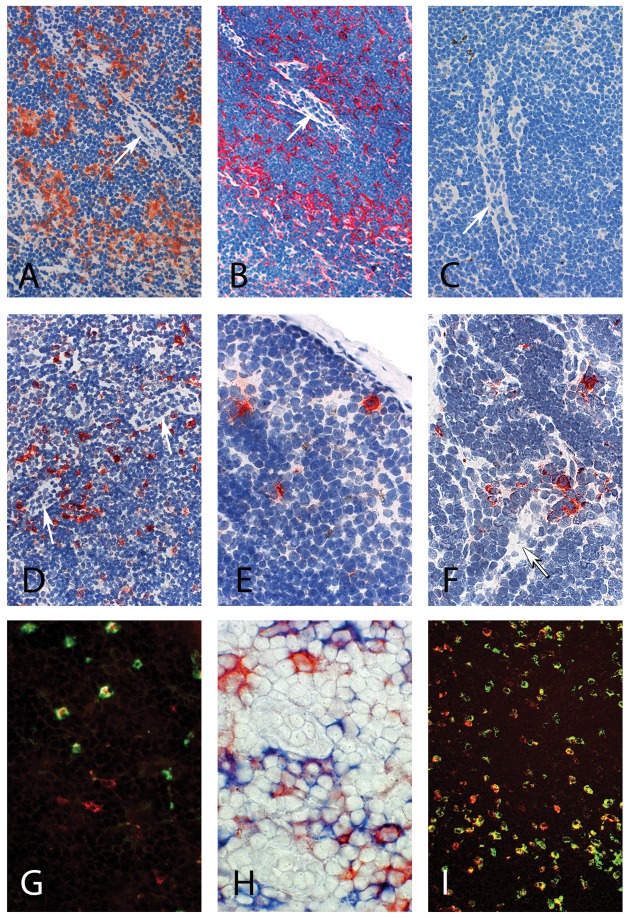
In vitro and in vivo binding of anti-DEC-205-p41 to DCs located in rhesus macaque lymph nodes. (A–C) Frozen sections from control lymph nodes were incubated with 3G9-p41 followed by anti-IgG (brown in A) or anti-HIV capsid mAb (red in B) staining. No immunolabeling was seen when anti-HIV capsid mAb was omitted (C). (D–H) 48 h after s.c. application of 3G9-p41, draining (D; E, marginal sinus) and contralateral (F) lymph nodes were removed and sections were stained with anti-HIV capsid antibody (red). The high endothelial venules as characteristic structures of the T-cell area of the lymph node are highlighted by arrows. Targeting of DCs by 3G9-p41 was verified by double labeling of the sections with CD1a (red in G, blue in H), which is expressed by immature DCs, or CD83 (red in I), typically expressed by mature DCs, and anti-p24 (green in G and I, red in H).

**Figure 3:**
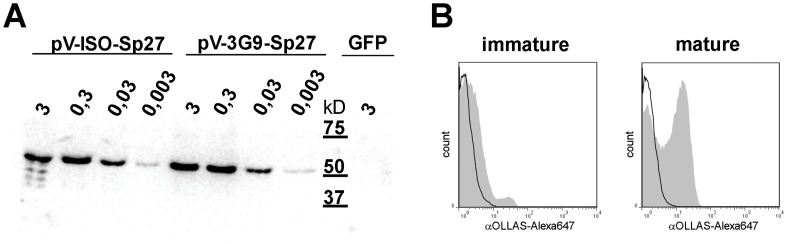
Expression and characterization of the antigen and verification of binding to rhesus macaque DCs. 293 T cells were transiently transfected with grading doses (from 3 to 0,003 µg) of scISO-p27 or scDEC-p27 expressing plasmids (pV-scISO-p27 or pV-scDEC-p27). Additionally, 293 T cells were transfected with 3 µg of a GFP-expressing plasmid as negative control. Supernatants were harvested 48 h after transfection and secreted fusion proteins were detected by Western Blot analysis to confirm comparable expression levels (A). Monocyte-derived rhesus macaque DCs were incubated with supernatants of transfected 293 T cells, and bound fusion proteins were visualized by using an Alexa647-labeled α-OLLAS antibody. Subsequent FACS-analyses are shown for scDEC-p27 (filled grey histogram) and scISO-p27 (open black histogram) for immature and mature DCs (B).

To generate a single chain antibody to rhesus macaque DEC-205, we first explored whether 3G9, a monoclonal antibody (mAb) generated by immunization of human immunoglobulin transgenic mice with human DEC-205 [5], cross-reacts with the macaque protein. Lymph node sections from macaques not previously exposed to HIV or SIV antigen were incubated with 3G9 coupled to the HIV-p41 Gag fragment (3G9-p41). This mAb consists of human IgG1 constant domains and a truncated HIV p55 protein, and subsequent incubation with antibodies against these antigens revealed binding of 3G9-p41 to large cells with abundant cytoplasm, which were located in the T-cell region as indicated by the presence of high endothelial venules (Fig. 2 A and B). No immunolabeling was seen when anti-p24 (Fig. 2C) or 3G9-p41 (data not shown) was omitted (Fig. 2C). Thus, 3G9 coupled to the HIV-p41 Gag fragment recognizes antigens, most likely expressed by myeloid DCs, in monkey lymphoid tissue.

To further explore possible in vivo targeting, 3G9-p41 was injected s.c. in the groin of three rhesus macaques. After 48 hours, a lymph node draining the injection site as well as a contralateral lymph node were removed. After staining of lymph node sections by using an anti-HIVp41 mAb, labeled cells were found scattered in both lymph nodes (Fig. 2D and F) as well as in the marginal sinus of the draining node indicating migrating cells, which is typical for DCs (Fig. 2E). Comparable results were found for all three monkeys. The findings suggest systemic distribution of the injected 3G9-p41 by the targeted cells. As observed before in vitro, the target cells were mainly localized in the T-cell zone as indicated by the presence of high endothelial venules. This finding and the cellular morphology of the labeled cells strongly suggested that interdigitating myeloid DCs were targeted by the antibody. To determine the lineage of the immunostained cells, we performed double staining with mAbs recognizing molecules typically expressed by immature (CD1a) or mature DCs (CD83). Immunofluorescence and immunohistochemistry revealed co-localization of 3G9-p41 with both CD1a^+^ and CD83^+^ cells (Fig. 2G–I).

To construct a single chain antibody with the binding specificity of 3G9, we cloned the reading frames of the variable heavy and light chain of 3G9 in frame to the coding region of SIV p27 capsid and tagged with the OLLAS epitope [20] (scDEC-p27). As a control, a single chain antibody to an irrelevant antigen was fused to SIV p27-OLLAS (scISO-p27). Since one critical step for our vaccination is the comparable expression and secretion of our fusion proteins from transduced muscle cells, we transfected 293 T cells with graded doses of the expression plasmids to ensure comparable expression levels over a broad concentration range. In the supernatant of the transfected cells, the secreted fusion proteins of the expected size were detected in comparable amounts for both plasmids by Western Blot analysis (Fig. 3A). To confirm specific binding to DEC-205, immature and mature rhesus macaque DCs were generated from peripheral blood monocytes, incubated with the supernatants of 293T cells transfected with scDEC-p27 or scISO-p27, and subsequently stained with a mAb to OLLAS. We observed binding of scDEC-p27, but not of scISO-p27, to mature and to a lesser extent to immature DCs (Fig. 3 B), which express less DEC-205 than mature DCs [21]. This mirrored binding experiments on human DCs and CHO cells stably expressing huDEC205 and confirmed the cross-reactivity of the single-chain antibody (data not shown).

### Immunogenicity of DNA encoding DEC-205-targeted antigen

To directly evaluate the effect of targeting antigens encoded by DNA vaccines to DEC-205 on the immunogenicity, one group of six rhesus monkeys (group A) was immunized with scDEC-p27 while group B received scISO-p27. Both groups were immunized twice (week 0 and 8) at a dose of 0.1 mg DNA by intramuscular electroporation. Two additional groups (group C and D) received 1 mg of scDEC-p27 twice by conventional intramuscular injection. In group C, the DNA vaccine was co-injected with poly ICLC as adjuvant.

**Figure 4:**
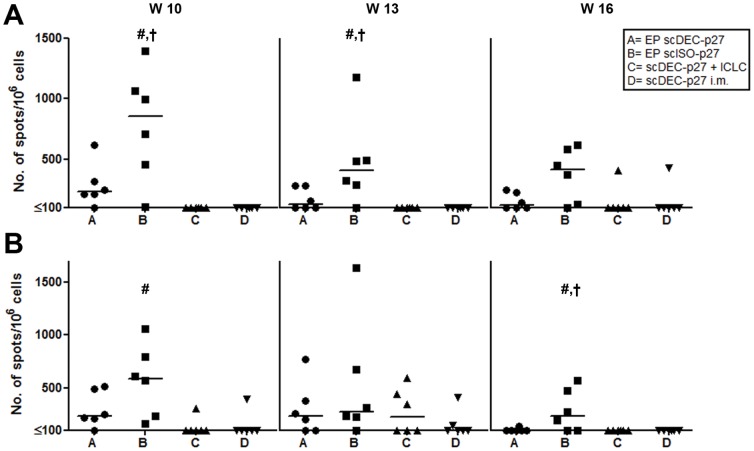
Induction of SIV-specific IFN-γ secreting cells by control but not by DEC-205 targeting DNA. At weeks 10, 13, and 16, PBMCs were stimulated for 17–20 h with either AT2-inactivated SIV (A) or a Gag-specific peptide pool (B), and numbers of IFN-γ producing cells were determined in ELISPOT assays. Numbers of spots per 10^6^ cells are presented for each animal and each point in time (line, median; #, p<0.05 compared with C and D, †, p<0.05 compared with A; one-way ANOVA followed by Bonferroni Post test).

Two, five and eight weeks after the second immunization, PBMCs were stimulated with aldrithiol-2-inactivated SIV (AT2-SIV) (Fig. 4 A) or a pool of selected peptides spanning the SIVgag polyprotein (Gag-peptide) (Fig. 4 B) to determine the T-cell response in an IFN-γ ELISPOT assay. Two weeks after the second immunization, both groups vaccinated by electroporation showed consistently moderate to high numbers of IFN-γ secreting cells which then gradually decline over time. The mean responses were significantly higher in the group primed with the non-targeting DNA (group B) compared to those observed in group A (Gag-peptide stimulated cultures at week 16, AT2-SIV stimulated cultures at weeks 10 and 13; p<0.05 by One-way ANOVA). Although groups C and D had received a 10-fold higher dose of DNA than groups A and B, IFN-γ producing cells were only detectable occasionally after conventional DNA immunization with scDEC-p27 (Fig. 4).

**Figure 5:**
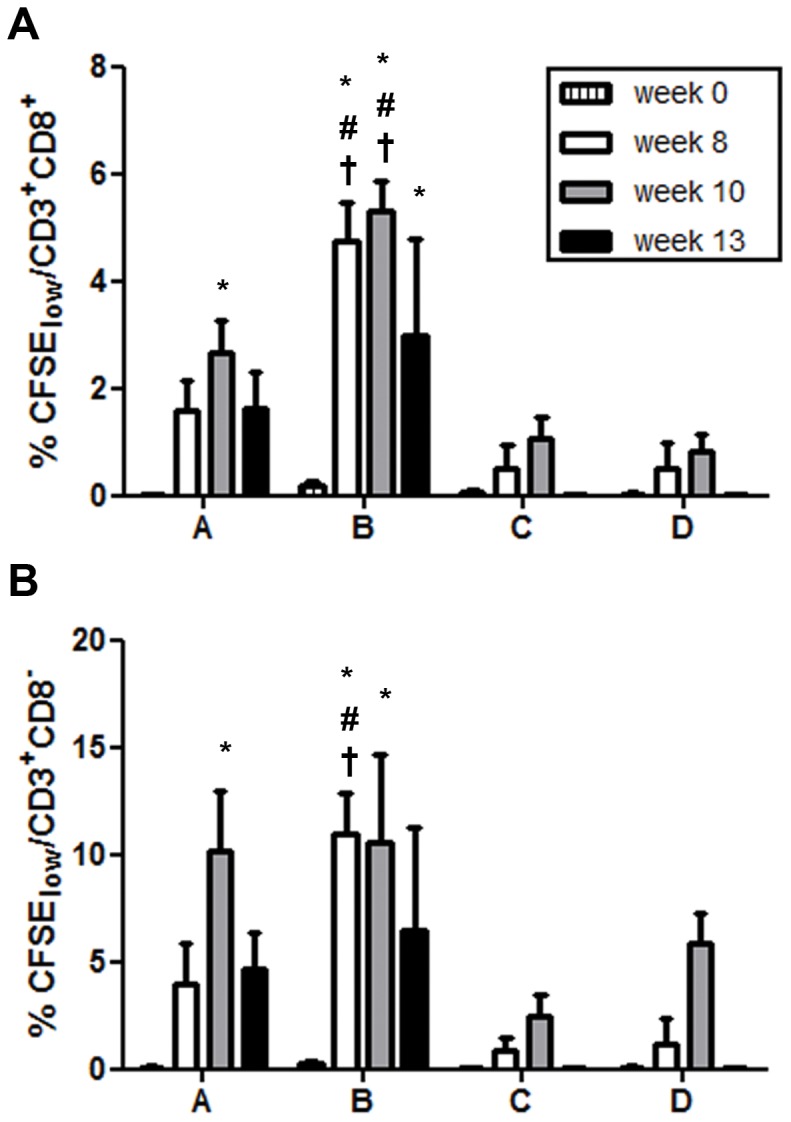
Development of proliferative CD4^+^ and CD8^+^ T-cell responses following DNA immunization. Before and at the indicated time points following immunization, CFSE-labeled PBMCs were incubated with AT-2 SIV or microvesicles. On day 7, T-cell proliferation was assessed as the percentage of CFSE^low^ CD4^+^ (gating on live CD3^+^CD8^−^, A) and CD8^+^ (gating on live CD3^+^CD8^+^, B) T cells. Data obtained with microvesicles were subtracted, and means and standard error of the means (SEM) are shown (#, p<0.05 compared with C and D, †, p<0.05 compared with A; one-way ANOVA followed by Bonferroni Post test; *, p<0.05 for differences to baseline; two-way ANOVA).

We determined SIV-specific proliferative responses in CFSE-dilution assays. Strongest responses were seen in the animals from group B where proliferation of CD4^+^ and CD8^+^ T cells was detected as early as week 8 and furthermore at weeks 10 and 13 after immunization (Fig. 5). Two immunizations were needed for the animals of group A to develop proliferative CD4^+^ and CD8^+^ T-cell responses, which already declined at week 13. In contrast, T-cell proliferative responses by the animals of groups C or D never exceeded significantly the values obtained at baseline (Fig. 5).

**Figure 6:**
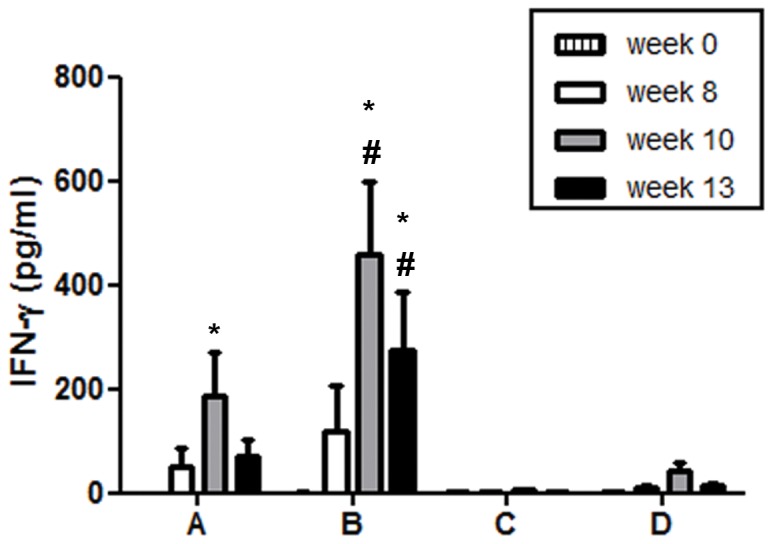
Analysis of cytokine secretion by PBMCs following SIV-restimulation. At the indicated time points, PBMC were incubated with AT2-inactivated SIV, microvesicles, or SEB as control. Culture supernatants were harvested after 48 h and concentrations of IFN-γ were determined by ELISA. Data obtained with microvesicles were subtracted, and means and SEM are shown (#, p<0.05 compared with C and D, †, p<0.05 compared with A; one-way ANOVA followed by Bonferroni Post test; *, p<0.05 for differences to baseline; two-way ANOVA). Substantial concentrations of IL-4, IL-10, or IL-17 were not detected in supernatants of SIV-stimulated cells derived from either group (data not shown).

We further characterized the T-cell responses by determining the concentrations of IFN-γ, IL-4, IL-10, and IL-17 in supernatants collected from re-stimulated PBMCs 48 h after setting up the assays. Cells from animals of group A produced significantly more IFN-γ at week 10 than before immunization and similarly those from group B monkeys at weeks 10 and 13 (Fig. 6). Consistent with the results of the IFN-γ ELISPOT and the CFSE dilution assays, we also detected significantly more IFN-γ in supernatants from cells derived from group B than from group A animals (Fig. 6). In contrast, we did not detect substantial amounts of IFN-γ in supernatants from cells derived from the animals of groups C or D. Notably we were unable to detect considerable amounts of IL-4, IL-10, or IL-17 in the supernatants from assays set up with PBMCs from either group of animals (data not shown).

**Figure 7:**
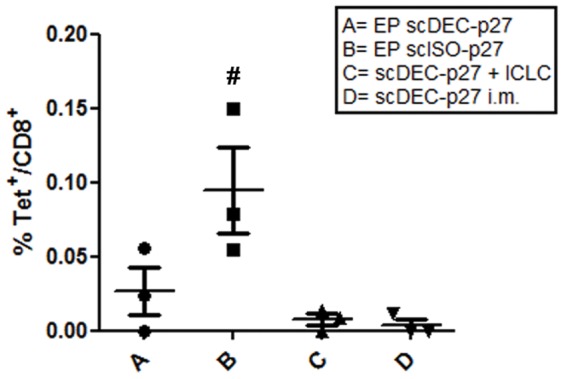
Tetramer analysis of antigen-specific CD8^+^ T-cell responses. Antigen-specific CD8^+^ T-cells were analyzed by tetramer staining for the immunodominant CM9 epitope at week 13. Lymphocytes from PBMC were stained for CD45, CD3, and CD8. The mean percentages of CM9-tetramer positive cells in CD8 T^+^ cells are shown for individual animals in each group (p<0.05 compared with C and D, †, p<0.05 compared with A; one-way ANOVA followed by Bonferroni Post test).

Similar observations were made using tetramer staining for the detection of SIV-specific CD8^+^ T cells. Since three animals of each group were Mamu-A*01 positive, we were able to identify CD8^+^ T cells specific for the immunodominant SIVgag CM9 epitope. Although the overall responses were rather low in the immunized animals, tetramer positive cells were mainly detectable in the blood of monkeys, which had received the DNA followed by electroporation (Fig. 7). Again, the non-targeting DNA tended to induce higher responses than DC-targeting DNA.

**Figure 8:**
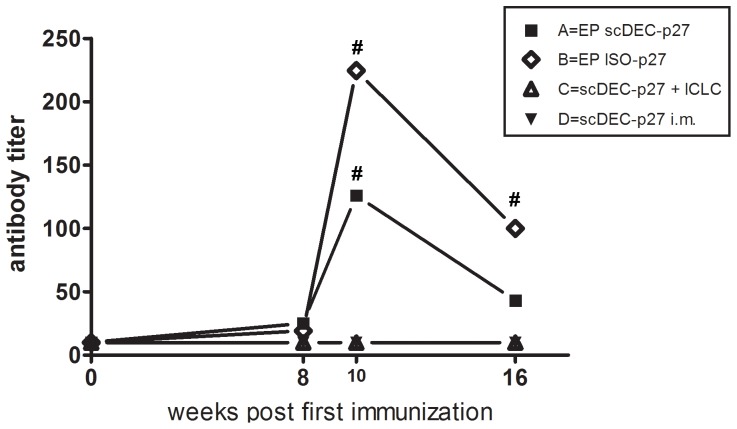
Development of humoral immune responses following DNA immunization. At the indicated time points, Gag-specific serum antibody titers were analyzed using an ELISA. Data are shown as geometric mean of six animals per group (#, p<0.05 compared with C and D; non-parametric ANOVA (Kruskal-Wallis) followed by a Dunns Post test).

The humoral immune response after vaccination was monitored by measuring Gag-specific antibodies. After two immunizations with DNA vaccines encoding either DEC-205 targeted or non-targeted antigen by electroporation, Gag antibodies were readily detectable in all animals of group A and B (Fig. 8), with approximately 2-fold higher antibody titers in group B. In contrast, conventional immunization with DNA encoding DEC-205 targeted antigens did not induce detectable levels of Gag antibodies even when poly ICLC had been used as adjuvant.

## Discussion

Our study aimed at improving the immunogenicity of DNA vaccines in primates by targeting the encoded antigen to an antigen-uptake receptor expressed by myeloid DCs, DEC-205 [22]. Since earlier mouse studies demonstrated the need for the simultaneous delivery of an adjuvant to generate robust T-cell responses after DC-targeted vaccination [4], [11], [12], [17], we either delivered the DNA by electroporation or injected it together with the TLR3-ligand poly ICLC that has previously been shown to augment protein-specific cellular immune responses in non-human primates [7], [8]. One of the most impressive improvements by DC-targeting DNA vaccines in mice were the high immunogenicity at rather low doses [18], which was the reason that suboptimal doses of DNA were used for the intramuscular injection and the electroporation approach. The failure of DC-targeting DNA, independent of the use of poly ICLC, to induce substantial immune responses after intramuscular injection might therefore be a consequence of low antigen expression levels in vivo. The low immunogenicity of this “classical” injection protocol is in line with previous reports on DNA vaccines, which resulted in poor CTL or antibody responses after two immunizations with comparable or even higher amounts of gag and env-expressing plasmids [23], [24]. The addition of poly ICLC could not enhance the immunogenicity of our DNA vaccine, although the immunization with DC-targeted protein plus poly ICLC as adjuvant has proven to be a successful strategy [4]–[8]. This might be a consequence of the delayed antigen expression after DNA delivery compared to protein vaccines, where poly ICLC and the targeted antigen are available at the same time. For DNA vaccines encoding DEC-targeted protein, it may be possible that the inflammatory response induced by poly ICLC within hours (i.e., maximum CXCL10 serum levels are detectable 18 h after s.c. injection [9], have already declined before appreciable levels of antigens are expressed. Thus, for a prolonged period of time following immunization, antigen would be produced in vivo in the absence of adjuvant. Subsequent strategies may therefore focus on other adjuvants, e.g., DC-activating cytokines, such as GM-CSF or Flt-3 ligand, that could be included in the DNA constructs and thereby would be present for the entire period of antigen expression. Co-expression of GM-CSF during DNA immunization has been shown to enhance cellular as well as humoral immune responses in monkeys [25], [26]. Furthermore, expression of Flt-3 ligand expanded DC-populations in monkeys and was shown to act together with a TLR-9 ligand as potent adjuvant for DNA vaccine resulting in strong cellular immunity to SIV [27].

In comparison to the conventional intramuscular injection, the delivery of DNA by electroporation proved to considerably enhance the immunogenicity of the injected DNA. This is in line with previous reports where the immunogenicity of DNA vaccines against a variety of infectious agents, e.g. HIV/SIV, HCV, or *Bacillus anthracis*, could be improved by applying electric pulses together with the DNA [28]–[32]. In our study, the non-targeting DNA elicited humoral and cellular responses, which were comparable to previous studies using DNA electroporation with comparable doses (100 µg–250 µg) [29], [33]. Although it is difficult to compare different vaccine studies due to differences in the antigens, dosages and application routes applied, our administration protocol seems to be well suited to generate antigen-specific immune responses in the non-human primate model. The response seems to be dominated by CD4 T cell responses and is in line with the study of Rosati et al, in which the immune response against the native form of Gag was also predominantly composed of CD4 cells [29]. However, targeting the antigen to DCs in rhesus macaques did not enhance the humoral immune response and reduced the cellular immune response. This is in sharp contrast to the observations in the mouse model [18]. Since our targeted and non-targeted antigens only differ in the specificity of the single chain antibody and showed comparable expression and secretion efficiencies, the reduced immunogenicity is most likely due to the targeting of DCs. Although we could not analyze antigen presentation directly in the macaque model, we could clearly demonstrate binding of the scAb to rhesus DCs comparable to human DCs. It has been previously shown that coupling of antigen to either DEC205-specific antibodies or scAb lead to efficient antigen uptake and presentation by human DCs [34], [35]. Therefore we hypothesize that differences in the antigen presentation by DCs after DEC-targeting and non-targeting DNA immunization are the underlying reason for the reduced immunogenicity we observed in rhesus macaques. Since immunization with DEC-205-targeted antigens in the absence of DCs maturation stimuli has been shown to induce tolerance rather than immunity in mice [10], [11], [15], [16], it is a plausible hypothesis that comparable processes were induced in non-human primates as well. Therefore, the induction of regulatory T-cells or depletion of antigen-specific T-cells could be potential consequences of DC-targeting in the absence of a strong adjuvant resulting in reduced immune response or tolerance. While there is no direct evidence for this in non-human primates so far, one could test this hypothesis by injecting a related protein antigen after immunizing monkeys with DC-targeting DNA. If tolerance had been induced by the DNA injection, one would expect considerably lower (if any) immune responses in pre-injected animals, and subsequent experiments should address this important issue.

In conclusion, although we did not observe enhanced antigen-specific immune responses by targeting the antigen encoded by a DNA vaccine to DEC-205 in nonhuman primates, targeting of protein vaccines to DCs remains an attractive vaccine strategy. However, a better understanding of the in vivo requirements for DC-driven T-cell activation in primates and humans is needed to explore the full potential of DC-targeted vaccines and to turn the enhanced antigen presentation into potent and protective immune responses. Co-delivery of DNA encoding stimulatory molecules might be a promising avenue for further investigation of DC-targeting DNA vaccines.

## Materials and Methods

### Animals

To initially prove binding of the complete anti-DEC205 antibody in non-human primates three young adult colony-bred male rhesus macaques (*Macaca mulatta*) from China were used. For the DNA immunization study twenty-four young adult colony-bred Indian-origin rhesus macaques of either sex were assigned to four experimental groups with six animals each. All animals were housed at the German Primate Center under conditions according to the German Animal Welfare act complying with the European Union guidelines on the use of non-human primates for biomedical research. This includes measures of animal welfare and amelioration of suffering in all work such as a 12∶12 light dark schedule, provision of monkey biscuits supplemented with fresh fruit twice a day and constant water access. Additionally, the monkeys were kept under permanent medical supervision. In cases of suffering predefined by a scoring system on termination criteria, monkeys were humanely killed. Both, the DEC205-binding study as well as the DNA immunization study were approved by an external ethics committee authorized by the Lower Saxony State Office for Consumer Protection and Food Safety and performed with the project licenses 33.9.42502-04-072-08 and 33.9.42502-04-017/07, respectively, issued by the same State Office. All animals were seronegative for SIV, simian retrovirus, and T-cell leukemia virus. Major histocompatibility complex (MHC) class I allele genotyping of the macaques of Indian origin was carried out as described before [36], [37]. 12 macaques were identified carrying the Mamu-A*01 allele and three each were allocated to each study arm. For collection of blood samples animals were sedated i.m. with 10 mg ketamine per kg body weight. For deeper anesthesia required for immunization or lymph node removal a mixture of ketamine, xylazine and atropine was used.

### Construction and characterization of DNA vaccines

The fusion of the single chain antibodies scDEC and ISO to HIV antigen was previously described [17], [18]. A codon-optimized plasmid encoding the control antibody fused to SIVgag (pV-scISO-gag) was obtained from Geneart. To generate p27-OLLAS containing fusion proteins, the sequence was amplified by PCR including the OLLAS sequence [20] in the antisense primer of p27. The PCR product was cloned in place of full-length SIVgag resulting in pV-scISO-p27. Furthermore, the sequence of the scDEC single chain based on the monoclonal antibody 3G9 [5] was amplified by PCR and replaced the sequence of the control antibody. The resulting plasmid was referred to pV-scDEC-p27. Both plasmids were based on the pVAX backbone plasmid (Invitrogen) where the antigen expression is driven by a CMV promoter. For in vivo studies, plasmids were purified with the NucleoBond® PC 10.000 EF Kit (Macherey-Nagel, Düren, Germany) according to the manufacturer's protocol and then tested for endotoxin levels with the LAL quantification assay (Cambrex Bio Science, Verviers, Belgium) confirming that the dose used for immunization contained less than 0.1 EU (Endotoxin Units).

To verify equal expression levels of the two plasmids, 293 T cells were transiently transfected in the presence of polyethlyeneimine (PEI) as described elsewhere [38] and supernatants were harvested after 48 h and subjected to western blot analysis. The secreted fusion proteins were detected by the combination of α-OLLAS [20] and rabbit-α-ratIg-HRP antibodies (Dako). Moreover, supernatants were tested for their binding capacity to DEC-205 expressed by rhesus macaque DCs. Monocyte-derived immature and cytokine-activated, mature DCs were generated as previously described [39] and incubated with supernatants of scDEC-p27 or scISO-p27 transfected cells for 30 min at 4°. Bound fusion proteins were stained by an Alexa647-labeled α-OLLAS antibody and the cells were analyzed by flow cytometry on a FACScalibur® (BD Bioscience).

### Characterization of 3G9 antibody binding in vitro and in vivo

Lymph nodes of non-treated control macaques were selected from our files. Five µm thick sections were prepared with a cryostate. The sections were fixed in 4% paraformaldehyde and incubated with the fusion protein 3G9-p41 followed by incubation with either anti-human IgG or with anti-p24 of HIV (both from Dakocytomation Hamburg, Germany). Binding of the polyclonal antibody was detected by incubation with StreptABComplex/HRP (code K0391; Dako, Hamburg, Germany) using 3-amino-9-ethylcarbazole (Sigma-Aldrich, Deisenhofen, Germany) as the substrate. Binding of anti-p24 was visualized using the alkaline phosphatase anti-alkaline phosphatase (APAAP) reaction with Fast red as substrate. The sections were counterstained with haemalaun and mounted.

To test for binding in vivo, three rhesus monkeys received 250 µg 3G9-p41 in 1.3 ml saline in the right groin. Lymph nodes from the same and the contralateral region were surgically removed 48 h after antigen application and partially snap frozen in liquid nitrogen and kept at −80°C until use. Cryostat sections were stained with anti-p24 as described above and counterstained with haemalaun. Double labeling for lineage characterization of 3G9-p41^+^ cells was performed by an overnight incubation with either anti-CD1a for immature (NeoMarkers, Freemont, CA; clone 010) or anti-CD83 for mature DCs (Novocastra Laboratories, Newcastle-upon-Tyne, United Kingdom). Isotype matched secondary antibodies labeled with FITC or TRIC were applied. The images were taken with an AxioImager M1 microscope (Carl Zeiss, Jena Germany) running an AxioVision rel.4.6.

### Immunizations

The different DNA vaccines were delivered twice spaced eight weeks apart as follows. Group A received scDEC-p27 DNA via a “needle style” electroporation (EP) device (TriGridTM Delivery System, Ichor Medical Systems, San Diego, CA). A 1 ml-syringe with 250 µl DNA solution containing 0.1 mg DNA was loaded into the EP device and adjusted to an injection depth of 15 mm as advised by the manufacturer. Then syringe and device were applied in tandem into one quadriceps muscle and the DNA was manually delivered intramuscularly by rapid bolus injection. After 10 seconds the electrical pulse was initiated using electroporation conditions as previously described [28]. The same procedure was performed on the other quadriceps muscle. Group B served as a control for the DEC-205-targeting and received non-targeted scISO-p27 DNA at the same dose and by the same approach as described for group A. Group C was given 1 mg scDEC-p27 DNA mixed with 2 mg of poly ICLC (Hiltonol, Oncovir, Washington, D.C.), by the intramuscular (i.m.) route in a final volume of 1.3 ml delivered in equal volumes into each hamstring muscle. Finally, group D received scDEC-p27 DNA under the same conditions as group C, but without adjuvant.

### Detection of cellular immune responses

Blood samples were drawn before and at regular intervals after immunization to measure SIV-specific cellular and humoral immune responses. To measure SIV-specific IFN-γ secreting T-cells, an ELISPOT assay was performed as described [40]. For antigenic stimulation SIV Gag peptides (EVA7066.1-16, NIBSC) and aldrithiol-2 (AT-2)-inactivated SIV (ARP1018.1, NIBSC, lot # P4002), the latter kindly provided by the National Cancer Institute (Frederick, MD) and distributed through NIBSC, Centre for AIDS reagents, UK, were used. As an SIV-unrelated peptide control stimulus, a pool of six 20-mer peptides derived from the gHCV NS3 gene was included [41]. Inactivated microvesicles derived from SUP-T1 cells (ARP1018.2, NIBSC, lot # P3824) served as control stimulus for AT-2 SIV.

Proliferation assays were set up with carboxyfluorescein diacetate succinimidyl ester (CFSE, Invitrogen/Molecular Probes, Karlsruhe, Germany)-stained PBMCs as described [9]. Briefly, PBMCs at 1×10^7^ cells/ml were stained with 0.25 µM CFSE in pre-warmed PBS for 15 min at 37°C, washed in medium, incubated in pre-warmed medium for another 30 min, and washed again. The cells were cultured at 1×10^5^ PBMCs/well in 96-well round-bottom trays (Nunc) in the presence of AT-2 SIV (300 ng p27/ml) or unspecific microvesicles identically prepared (ARP1018.2, concentration adjusted to protein content of AT-2 SIV). PBMCs in medium alone or stimulated with 5 ng/ml staphylococcal enterotoxin B (SEB; Alexis Corp., Lausen, Switzerland) served as controls. All conditions were set up in triplicates and cultures were incubated at 37°C and 5% CO_2_. On d 7, cells were harvested and washed in PBS/5% FCS/0.05% sodium azide, stained with anti-CD3 PE- and anti-CD8 PerCP-conjugated mAbs, washed, and fixed. T cell proliferation was assessed as the percentage of CFSE^low^ cells, gating on live CD3^+^CD8^+^ or CD3^+^CD8^–^ cells. Likewise, stimulated and unstimulated PBMCs were incubated for 48 h, supernatants were harvested, and frozen at −80°C for analyses of cytokine concentrations.

Cytokine concentrations in cell culture supernatants were measured using ELISA kits for monkey IFN-γ, IL-4, and IL-10 (all U-Cytech, Utrecht, The Netherlands) and for human IL-17 known to cross-react with monkey IL-17 (eBioscience, NatuTec, Frankfurt/Main, Germany) [9].

MHC class I tetramer staining of SIV-specific CD8^+^ T cells was carried out for the Mamu-A*01-positive macaques. 50 µl of whole blood was incubated for 30 min with the phycoerythrin (PE)-conjugated tetramer Mamu-A*01 Gag181–189 (CM9, Beckman Coulter, Krefeld, Germany) and the BD Biosciences (Heidelberg, Germany) monoclonal antibodies anti-CD3 Alexa700 (clone SP34-2) and anti-CD8 AmCyan (clone SK1). Following surface staining, blood samples were treated with FACS lysing solution (BD Biosciences). Flow cytometric analysis was performed using a BD LSRII flow cytometer (BD Biosciences) and the list-mode data files were analyzed using FlowJo Version 8.7 (Tree Star).

### Detection of humoral immune responses

To determine humoral SIV-specific responses, a standard ELISA for the detection of antibodies against the SIV polypeptides gp130 SU and p27 CA [42] in a limiting-dilution format was employed. Recombinant SIVgp130 (EVA670, NIBSC) and SIVp27 (EVA643) were kindly provided by via NIBSC, Centre for AIDS reagents, UK.
